# Giant fibroepithelial stromal polyp of the vulva: largest case reported

**DOI:** 10.1186/1750-1164-7-8

**Published:** 2013-07-10

**Authors:** Obianuju Sandra Madueke-Laveaux, Radhika Gogoi, Gary Stoner

**Affiliations:** 1 Obstetrics-Gynecology, Geisinger Medical Center, Danville, PA 17821, USA; 2 Gynecologic Oncology, Geisinger Medical Center, Danville, PA 17821, USA

## Abstract

**Background:**

Fibroepithelial stromal polyps are site-specific mesenchymal lesions that are commonly found in the vulvovaginal region in premenopausal females. These polyps usually are less than 5 cm in diameter and are most commonly identified during routine gynecological examination. Although the stromal polyp is benign, its differential diagnosis includes some malignant vulva lesions making it critical to ensure that an accurate pathologic diagnosis is made.

**Case:**

We present a case of a 21 year old female with a giant fibroepithelial stromal polyp of the vulva. Upon review of the literature this is the largest reported fibroepithelial stromal polyp to date.

**Conclusion:**

Fibroepithelial stromal polyps can grow as large as 390 grams and can be 18.5-cm in diameter. Microscopic evaluation of the polyp is critical in the exclusion of malignancy with this diagnosis.

## Introduction

Fibroepithelial stromal polyps are a type of mesenchymal lesion that typically occur in women of reproductive age. These polyps are site-specific and have a predilection for the vulvovaginal region. They are most frequently found in the vagina. Infrequently they occur on the vulva and cervix and rarely are found in extra-genital sites. These polyps are hormone sensitive and most commonly occur in pregnancy. However they can also be seen in premenopausal females who are on hormone replacement therapy. These lesions typically do not grow larger than 5-cm in diameter and are found incidentally during routine gynecologic exams [1]. They can be polypoid or pedunculated and are usually solitary. Symptoms usually include bleeding, discharge and general discomfort with sensation of a mass. There are a few reported cases of giant fibroepithelial stromal polyps of the vulva. Bozgeyik et al. discuss the ultrasound and computed tomographic findings in a 15-cm fibroepithelial polyp [2]. Orosz et al. report a case of recurrent giant fibroepithelial polyp of the vulva in association with congenital lymphedema [3]. The initial polyp in the latter case was 10-cm in diameter. This case is unique because the polyp in our patient measured 18.5-cm in its widest diameter.

## Case

A 21 year old nulliparous female presented to the emergency department (ED) with the complaint of right lower quadrant abdominal pain. During her ED course, she admitted that her real reason for presentation was for examination of a mass that had been growing on her right labium. Following a brief physical examination by the ED physician, the gynecology team was consulted and verification was made of a right labial mass. The patient admitted that she first noticed a marble-sized “bump” on her right labium about six months earlier. The bump increased in size over the course of the six months until its current size on presentation. She reported being too embarrassed and afraid to address the growth but due to the polyp’s burdensome size she was forced to present for evaluation. She was on day two of her menstrual cycle and denied any nausea, vomiting or any constitutional symptoms. The patient denied any significant medical or surgical history. She denied any history of sexually transmitted disease or gynecology-related surgery. She reported a history of regular menstrual cycles with a menstrual index as follows - menarche at age 10, 30 day cycle intervals and 4 to 6 day cycle lengths with moderate flow. She had not been sexually active for over a year and was on no contraception. She was a non-smoker and denied alcohol or drug use. Her physical exam was remarkable only for a large, non-tender, skin colored, grapefruit-sized ulcerating pedunculated mass extending from the right labium majus. Transvaginal ultrasound showed normal anatomy of the uterus and ovaries but also described a broad-based encapsulated soft tissue mass of the right labium at the level of the clitoris. The patient was discharged home for follow up with outpatient Gynecology-Oncology. Physical exam by the oncologist again verified a right labial pedunculated mass. The stalk was noted to be vascular with an area of ulceration over the serosal surface of the polyp. There was no increase in the size of the mass with valsalva. An in-office procedure to amputate the polyp was completed at the oncologist’s office. The base of the mass was infiltrated with Xylocaine, 3 large Kelly clamps were placed across the base and the mass was excised. The pedicle was ligated with 0 Vicryl suture and adequate hemostasis was obtained. The patient received Percocet for pain control with instructions for a 1- week follow up appointment. At her follow up appointment the vulva was noted to be healing well and the stalk was retracted. The pathology report returned as a giant fibroepithelial stromal polyp and she was advised to return to her routine gynecologist for continued surveillance.

## Histopathology/discussion

**Figure 1:**
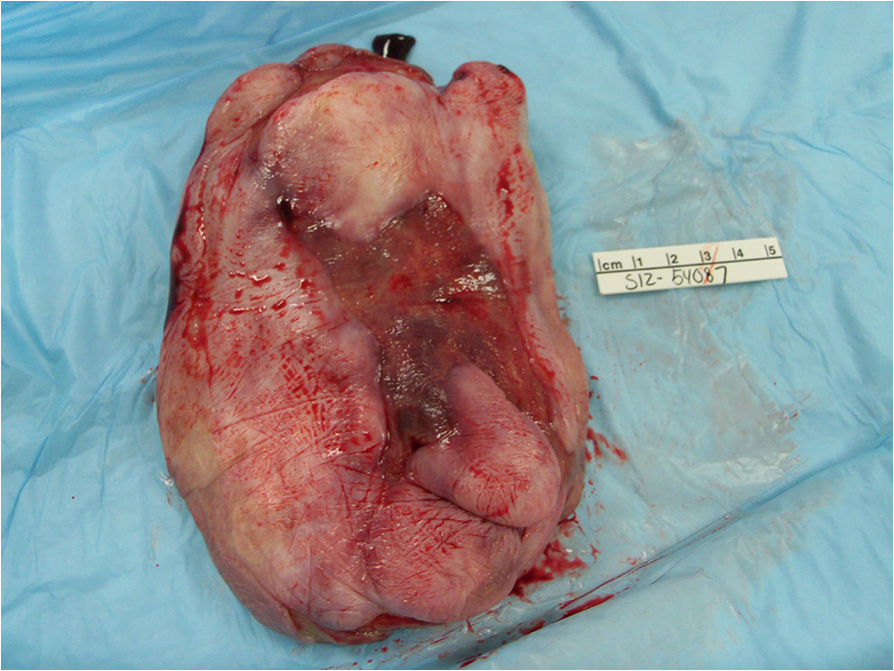
Ulcerating surface of the polyp.

**Figure 2:**
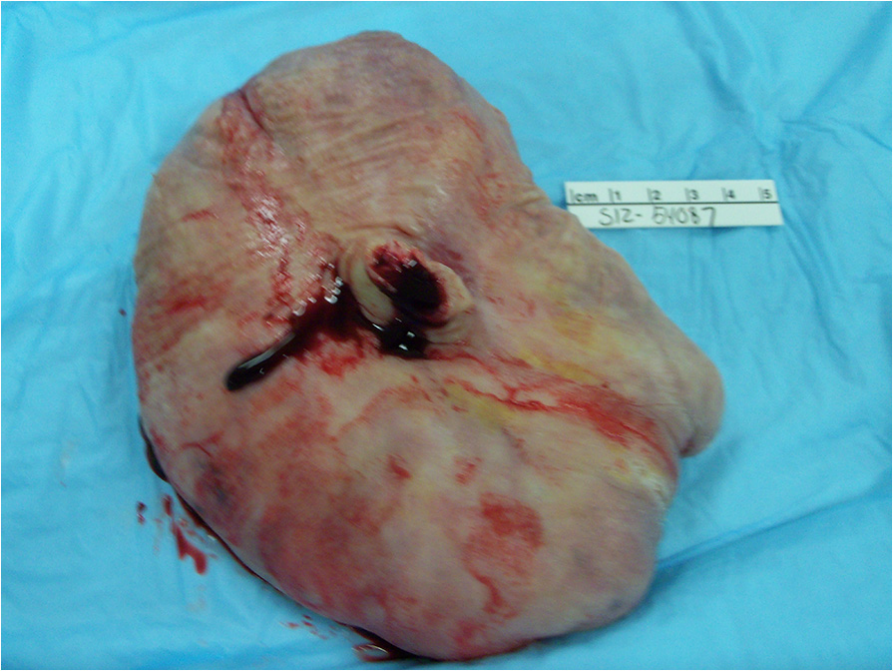
Centrally located stump of amputated stalk with cross-section of thick-walled vessel.

Fibroepithelial stromal polyps usually occur in women of reproductive age. They vary in size but are commonly polypoid and exophytic. The margins of these polyps merge with normal tissue and their vascular supply is usually thick-walled with a central core. Below are the gross pictures of the polyp in our case (Figures 1 and 2).

Malignancy must be excluded in every diagnosis of fibroepithelial stromal polyp. The characteristic morphologic features as described above should serve as a guide in making this distinction. However, sarcomas may be grossly similar in appearance to fibroepithelial stromal polyps and for this reason microscopy is critical for final diagnosis. Microscopically, the most characteristic feature of a fibroepithelial stromal polyp is the presence of stellate and multinucleate stromal cells which are usually identified near the epithelial-stromal interface [1]. The stromal cells of the polyp may also be positive for desmin, actin, vimentin, estrogen and progesterone receptors. Sarcomas can be distinguished from even the most pseudosarcomatous examples of fibroepithelial polyps because they have identifiable lesion margins, homogeneous cellularity and lack stellate and multinucleate stromal cells near the epithelial-stromal interface.

**Figure 3:**
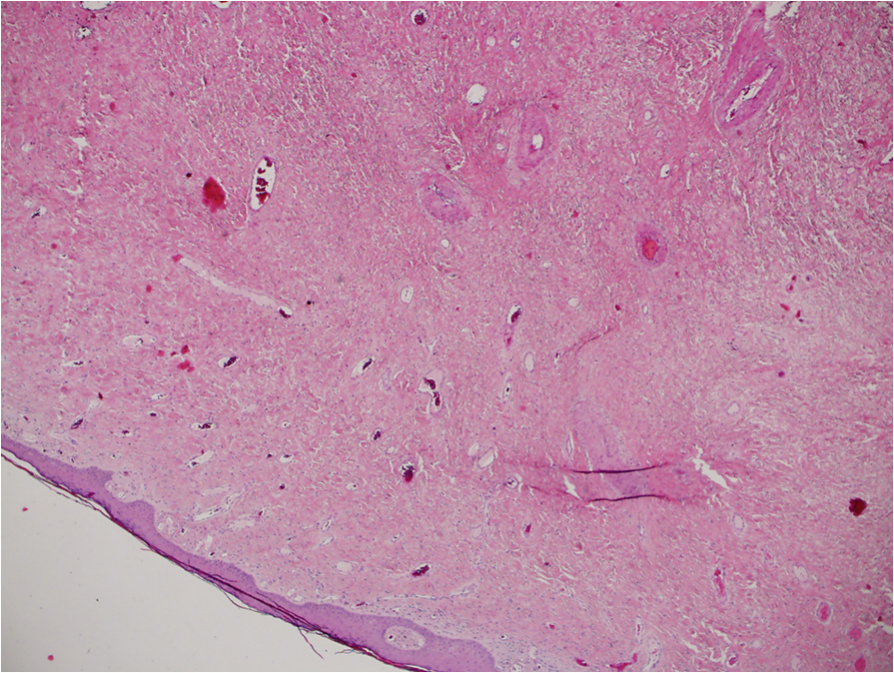
Histopathology showing myxoid stroma with land stellate cells extending to the epithelial-stromal interface.

**Figure 4:**
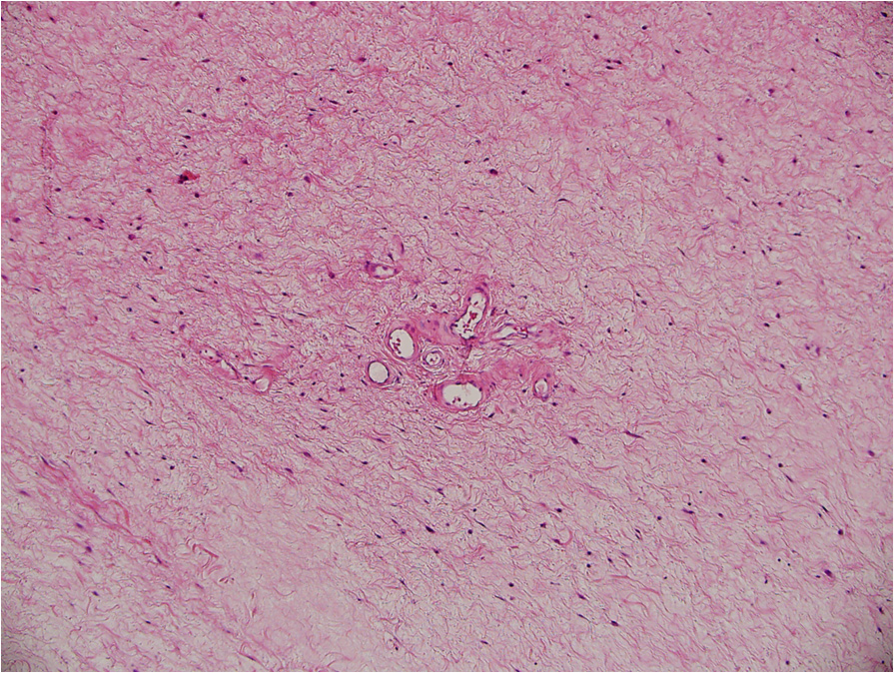
Slightly higher magnification of myxoid stroma with bland stellate cells.

Microscopic evaluation of the lesion in our case revealed stromal tissues with a fibrovascular supporting core. Focally, the stroma was myxoid with bland stellate cells extending up to the epithelial stromal interface. A section through the stalk of the polyp showed squamous epithelium surrounding a fibrovascular stalk with numerous thick walled vessels (Figures 3 and 4).

**Figure 5:**
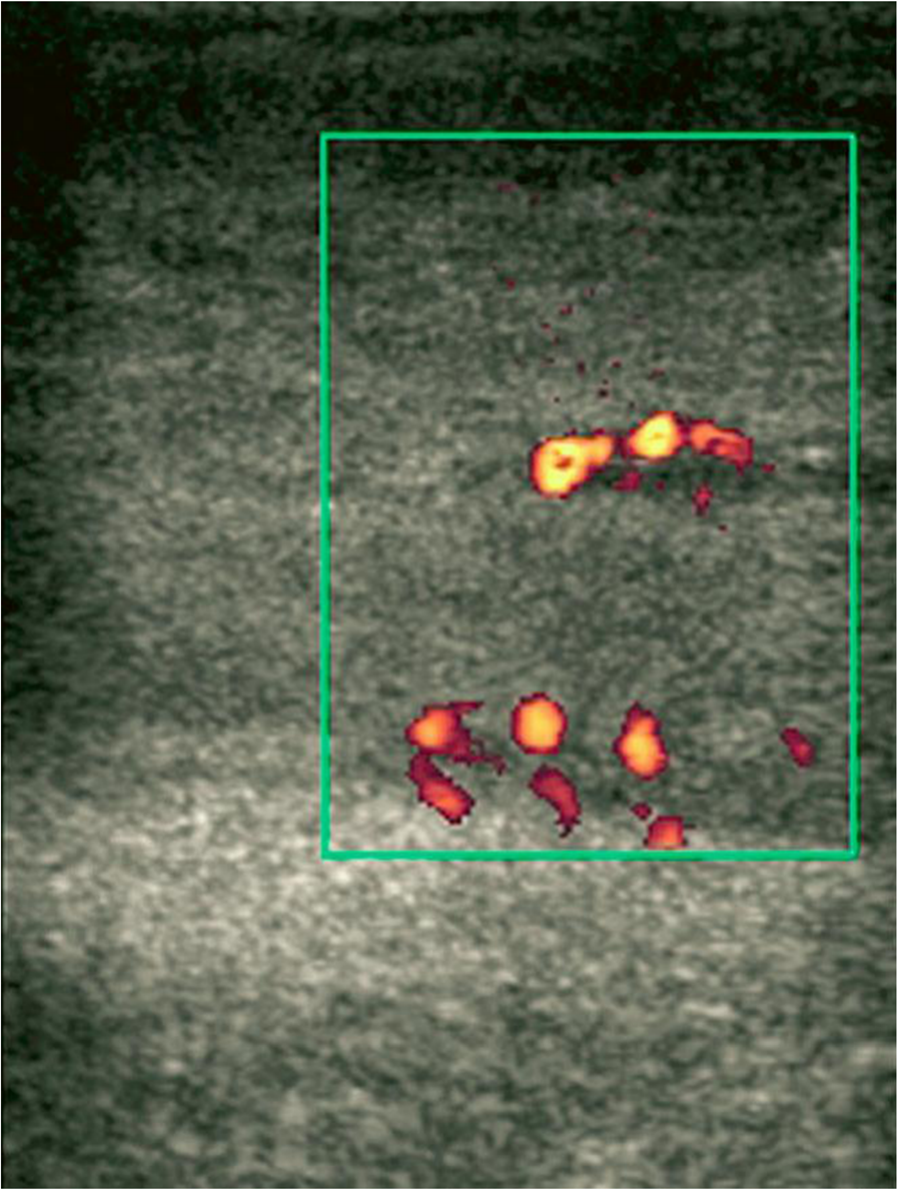
**Power Doppler Ultrasound image showing blood flow within a lesion**[2]**.**

In addition to histopathologic evaluation, imaging is important in the diagnostic work up of fibroepithelial stromal polyps. It allows for evaluation of blood supply and flow and demonstrates the origin and extent of the lesion. Although CT and MRI may be used, they are not as cost effective or widely available as ultrasonography [2]. As a result, ultrasonography is more suitable as a first line imaging tool. Ultrasonography also offers speed, capacity for dynamic exploration and ability to visualize the entire lesion in a single image (Figure 5).

The table below shows the clinical presentation and hisopathologic features of the site-specific mesenchymal lesions of the vulvovaginal region that must be considered in the differential diagnosis of fibroepithelial stromal polyps [4] (Table 1).

**Table 1:** **Differences in clinical presentation and histopathologic features**[4]

	**Aggressive angiomyxoma**	**Angiomyo-fibroblastoma**	**Cellular angiofibroma**	**Fibroepithelial stromal polyp**	**Superficial angiomyxoma**	**Prepubertal vulval fibroma**
**Age**	Reproductive age	Reproductive age	Reproductive age	Reproductive age	Reproductive age	Prepubertal
**Location/Configuration**	Deep seated, not polypoid	Subcutaneous	Subcutaneous	Usually polypoid, exophytic	Superficial subcutaneous, polypoid	Submucosal, subcutaneous
**Size**	Variable	Usually < 5 cm	Usually < 3 cm	Variable	Usually < 3 cm	Usually < 5 cm
**Margins**	Infiltrative	Well circumscribed	Usually well circumscribed	Merges with normal	Lobulated, distinct	Infiltrative
**Cellularity**	Paucicellular	Alternating hypercellular and hypocellular	Cellular	Variable	Hypocellular	Hypocellular
**Vessels**	Medium to large, thick-walled, hyalinized	Delicate, capillary-sized, numerous	Small to medium, thick-walled, hyalinized	Variable, usually large, thick-walled central core	Delicate, thin-walled, elongated	Small to medium-sized vessels
**Mitotic Index**	Rare	Usually uncommon	Variable, may be brisk	Variable	Usually uncommon	Uncommon
**Biomarkers**	Desmin positive, HGMA2 positive	Desmin positive, HGMA2 negative	CD 34 positive; Desmin, SMA variable	Desmin positive, HGMA2 negative	Desmin negative, HGMA2 negative	CD 34 positive; desmin, S-100 negative
**Clinical course**	30% local destructive recurrence	Benign	Benign	Benign, rare recurrence	30% local nondestructive recurrence	Benign, may recur

Botryoid Embryonal rhabdomyosarcoma is another differential that should be considered. However, these tumors are typically diagnosed in pre-pubertal females and they lack the characteristic hypercellular subepithelial layer and specific markers of skeletal muscle differentiation.

Although rare, fibroepithelial polyps can recur, especially if they are not completely excised. There are reported cases of such recurrence [5]. There is also at least one reported case of growth of a giant cell fibroblastoma at the site of a previously excised stromal polyp [6]. As a result, all patients with this diagnosis should be followed long term and managed appropriately after initial treatment.

## Conclusion

Fibroepithelial stromal polyps are benign, mesenchymal lesions that typically occur in women of child-bearing age. These polyps have a predilection for the vulvovaginal region and although they typically do not exceed 5 cm in diameter they sometimes can grow as large as 390 grams and 18.5-cm in diameter as seen in our presenting case. Although benign, the polyp mimics some more serious and malignant growths in appearance and as a result, microscopic evaluation of the polyp is critical in the exclusion of malignancy.

## Consent

Written informed consent was obtained from the patient for publication of this Case report. A copy of the written consent is available for review by the Editor-in-Chief of this journal.

## Competing interests

The authors declare that they have no competing interests.

## Authors’ contributions

OSM-L interviewed the patient, performed the literature review and drafted as well as revised the manuscripts. RG performed the excisional procedure and proof read all editions of the manuscript. GS participated in the interview of the patient, proof read all editions and contributed to the flow of the manuscript. All authors read and approved the final manuscript.
